# Pan-cancer analysis reveals that G6PD is a prognostic biomarker and therapeutic target for a variety of cancers

**DOI:** 10.3389/fonc.2023.1183474

**Published:** 2023-08-03

**Authors:** Tao Zeng, Bin Li, Xin Shu, Jiahui Pang, Heping Wang, Xianghao Cai, Yingying Liao, Xiaolong Xiao, Yutian Chong, Jiao Gong, Xinhua Li

**Affiliations:** ^1^ Department of Infectious Diseases, Key Laboratory of Liver Disease of Guangdong Province, The Third Affiliated Hospital of Sun Yat-Sen University, Guangzhou, China; ^2^ Department of Infectious Diseases, The First People’s Hospital of Kashi Prefecture, Kashi, China; ^3^ Department of Laboratory Medicine, The Third Affiliated Hospital of Sun Yat-Sen University, Guangzhou, China

**Keywords:** G6PD, pan-cancer, survival analysis, tumor microenvironment, drug resistance

## Abstract

**Background:**

Despite accumulating evidence revealing that Glucose-6-phosphate dehydrogenase (G6PD) is highly expressed in many tumor tissues and plays a remarkable role in cancer tumorigenesis and progression, there is still a lack of G6PD pan-cancer analysis. This study was designed to analyze the expression status and prognostic significance of G6PD in pan-cancer.

**Methods:**

G6PD expression data were obtained from multiple data resources including the Genotype-Tissue Expression, the Cancer Genome Atlas, and the Tumor Immunity Estimation Resource. These data were used to assess the G6PD expression, prognostic value, and clinical characteristics. The ESTIMATE algorithms were used to analyze the association between G6PD expression and immune-infiltrating cells and the tumor microenvironment. The functional enrichment analysis was also performed across pan-cancer. In addition, the GDSC1 database containing 403 drugs was utilized to explore the relationship between drug sensitivity and G6PD expression levels. Furthermore, we also performed clinical validation and *in vitro* experiments to further validate the role of G6PD in hepatocellular carcinoma (HCC) cells and its correlation with prognosis. The R software was used for statistical analysis and data visualization.

**Results:**

G6PD expression was upregulated in most cancers compared to their normal counterparts. The study also revealed that G6PD expression was a prognostic indicator and high levels of G6PD expression were correlated with worse clinical prognosis including overall survival, disease-specific survival, and progression-free interval in multiple cancers. Furthermore, the G6PD level was also related to cancer immunity infiltration in most of the cancers, especially in KIRC, LGG, and LIHC. In addition to this, G6PD expression was positively related to pathological stages of KIRP, BRCA, KIRC, and LIHC. Functional analysis and protein-protein interactions network results revealed that G6PD was involved in metabolism-related activities, immune responses, proliferation, and apoptosis. Drug sensitivity analysis showed that IC50 values of most identified anti-cancer drugs were positively correlated with the G6PD expression. Notably, *in vitro* functional validation showed that G6PD knockdown attenuated the phenotypes of proliferation in HCC.

**Conclusion:**

G6PD may serve as a potential prognostic biomarker for cancers and may be a potential therapeutic target gene for tumor therapy.

## Introduction

Cancer, emerging as a global issue, is a major concern for human health and a significant financial burden on global public health (1). According to data from 183 nations around the world, cancer is now the leading cause of death and a major global public health burden (2). Although various anti-tumor methods, such as surgery, radiation, chemotherapy, and immunotherapy, have been widely employed in clinical practice. Some tumors continue to progress and the treatment of most tumors is still largely unsatisfactory. Due to the intricacy of tumorigenesis, pan-cancer analysis is frequently employed in tumor research uncovering the similarity and heterogeneity of various tumor genes and involved biological processes, providing insight into cancer treatment and prevention (3, 4). Pan-cancer analysis projects, such as the Cancer Genome Atlas (TCGA), are widely used to identify specific functional genes, facilitating detailed cancer gene research (5, 6).

The gene encoding G6PD is located on the long arm of the X chromosome (Xq18.1), which is 18.5 kb in length and consists of 13 exons and 12 introns (7). Generally, G6PD deficiency may be a possible explanation for anemia (7). However, the role of G6PD in tumors has garnered in-depth attention in recent years. Existing evidence has shown that the level of G6PD is increased in a range of tumor cells, such as urinary tract cancer, breast cancer, cervical cancer, hepatocellular carcinoma, bladder cancer, lung cancer, and ovarian cancer (8–14). And the expression level of G6PD is related to the overall survival of tumor patients (15).

G6PD is generally considered to be the rate-limiting enzyme of the cellular pentose phosphate pathway (PPP), and PPP will manufacture sufficient reducing capacity, such as NADPH, which reduces excessive oxidative stress, thereby enhancing cellular antioxidant defense (15). The activity of the G6PD enzyme is associated with several biological processes of tumors, including angiogenesis, signal transduction, cell proliferation, apoptosis, and metastasis to remote sites (16, 17). However, G6PD’s function in tumors is still unclear. In actuality, there is still a lack of G6PD pan-cancer analysis. For these reasons, further investigation into the molecular mechanisms underlying G6PD’s function in tumors is crucial for improving clinical outcomes.

Immunotherapy strategies have emerged as a promising cancer therapy. The tumor microenvironment (TME), which contains complex cellular and noncellular components as well as their interactions, can serve as a prognostic biomarker in diverse types of cancers (18). G6PD is ubiquitously expressed in mammalian immune cells and the immunological microenvironment is regarded as the “seventh hallmark” of cancer (19). TME is directly associated with the occurrence, proliferation, and metastasis of tumor cells and its characteristics can be used as markers to evaluate the response of tumor cells to immunotherapy (20). Therefore, it is crucial to explore potential immune biomarkers and identify targets for cancer immunotherapy.

In this study, to demonstrate the biological role of G6PD in cancer, we utilized multiple databases to perform a visual pan-cancer analysis of G6PD. We examined the variations in G6PD expression in 33 different types of cancer and assessed the prognostic significance of G6PD using public databases. We also investigated its relationships with immune cell infiltration and functional enrichment analysis. The findings of this study contribute to the understanding of the role of G6PD in tumors and highlight the notion that G6PD is closely associated with patients′ prognosis and may represent a promising target for future therapies.

## Materials and methods

### G6PD data processing

The TCGA database contains the sequence information of genes in various tumor tissues, and the Genotype-tissue expression (GTEx) database contains the differential expression of sample genes in various tissues (21). Using the University of California Santa Cruz (UCSC) data portal (https://xenabrowser.net/datapages/) (22), we obtained pan-cancer patient RNA-Seq and clinical data from the TCGA database.

### G6PD expression analysis

Due to the lack of normal tissue data in the TCGA database and to comprehend the genetic differences between carcinomatous and corresponding adjacent normal tissues, we obtained the normalized pan-cancer datasets TCGA and GTEx from the UCSC database and extracted the expression data for the G6PD gene in each sample. Additionally, based on the TIMER database (https://cistrome.shinyapps.io/timer), differences in gene expression levels were evaluated between various tumor tissues and corresponding adjacent normal tissues. All expression data were normalized through log2 conversion.

### Survival analysis and correlation between G6PD and clinical phenotypes

Survival data of patients were obtained from the TCGA database. We applied a univariate Cox model to evaluate the association between G6PD expression levels and the survival outcomes of patients. A P-value of 0.05 was considered statistically significant. Based on the G6PD median expression value, the cancer cases were divided into two subgroups, including the “low” group (expression levels < median) and the “high” group (expression levels ≥ median). Kaplan-Meier (KM) analysis was implemented to evaluate the association between the G6PD expression levels and patients′ prognosis (OS: overall survival; DFI: disease-free interval; PFI: progression-free interval; DSS: disease-specific survival) by the log-rank test, and a survival-associated forest plot was generated. Furthermore, we also investigated the correlation between G6PD expression and patients′ clinical phenotypes in several cancers visualized by R packages “limma” and “ggpubr”. Statistical significance was shown by the following annotations: *p < 0.05, **p < 0.01, ***p < 0.001, and ****p < 0.0001.

### Immune cell infiltration into the tumor microenvironment

TIMER (https://cistrome.shinyapps.io/timer) is a comprehensive bioinformatics tool to systematically assess the degree of immune infiltration in diverse cancers (23). Using the TIMER algorithm, we investigated the association between G6PD expression levels and the infiltration levels of 6 different immune cell types (CD4^+^ T cells, CD8^+^ T cells, B cells, neutrophils, dendritic cells, and macrophages). The correlation of G6PD expression with the immune infiltrating scores of these 6 immune cells was evaluated by Spearman′s correlation analysis. Furthermore, we obtained the Immune Score and Stromal Score of multiple cancers via the “estimate” R package (24). We then used Spearman’s correlation method to analyze the correlation of G6PD expression with the Immune Score and Stromal Score. All of the gene expression levels were log2 transformed.

### G6PD-related gene analysis

Gene set enrichment analysis (GSEA) is an enrichment method, which uses predetermined gene sets to explore their expression status (25). The gene sets “HALLMARK, C2, C5, C7” were downloaded from the GSEA website (https://www.gsea-msigdb.org/gsea/msigdb/index.jsp). Based on the median value of gene expression level, clinical samples were divided into high- and low-expression groups, and signaling pathways in the above gene sets were analyzed with the GSEA method. Significant enrichment results were presented based on NES (Net enrichment score), gene ratio, and P value. Gene sets with |NES|>1, p-value <0.05, and FDR q <0.25 were considered to be the threshold of GSEA. The R packages “limma”, “org.Hs.eg.db”, “clusterProfiler” and “enrich plot” were applied to visualize the results.

STRING (https://cn.string-db.org/) is a commonly used database for functional protein association analysis (26). We obtained the liver cancer gene expression data from the TCGA database and conducted Pearson correlation analysis with G6PD expression. Based on this analysis, we identified 462 genes that were significantly correlated (r>0.55, p<0.05). Next, we uploaded these genes to the String database for constructing a protein-protein interaction (PPI). The specific parameters used in STRING are as follows: “Species: Homo sapiens”, “Minimum required interaction score: medium confidence (0.400)”, “Meaning of network edges: evidence”, “Active interaction sources: experiments, databases, co-expression, neighborhood, gene fusion, co-occurrence”. Subsequently, the Cytoscape software (version 3.9.1) was used to visualize the PPI and we used the MCODE plugin to identify hub genes. Subsequently, Gene Ontology (GO) and Kyoto Encyclopedia of Genes and Genomes (KEGG) enrichment analyses were conducted for the 462 genes associated with G6PD.

### Analysis of G6PD drug resistance

GDSC (https://www.cancerrxgene.org) is the public cancer drug sensitivity genomics database (27), and a total of 403 drug data were downloaded from the GDSC database to explore the correlation between gene expression in 970 cancer cell lines and drug half-maximal inhibitory concentration (IC50) using Spearman′s correlation analysis. Subsequently, according to the median G6PD expression level, cell lines were divided into low and high-expression groups. The Kruskal-Wallis rank-sum test was used to analyze the IC50 value of six anticancer drugs commonly used in clinical practice.

### Molecular docking

Molecular docking was used to evaluate the potential targeting relationship between anticancer drugs and G6PD (28). Firstly, the 3D structure of the G6PD protein (PDB: 2BHL) was obtained from the RCSB PDB database (https://www.rcsb.org/). Then we dehydrated and removed the ligands by using PyMOL software. Additionally, we downloaded the small molecule structures of the top three anticancer drugs from the PubChem database(https://pubchem.ncbi.nlm.nih.gov/), including *Tanespimycin, Flavopiridol*, and *PHA-793887*. Subsequently, we used the online pocket predictor (29) (https://playmolecule.com) to detect the possible docking site. The maximum pocket to cover the ligand was chosen, with a grid box centered at (-0.81, 133.51, 5.68) Å and a volume of 487,872Å^3^. Finally, molecular docking was performed using DOCK4.2.6 software (https://autodock.scripps.edu/) and the results were visualized by PyMol software.

### Patients and tumor tissues

To further validate the association between G6PD expression levels and the survival outcomes of patients, a total of 77 paraffin-embedded, archived hepatocellular carcinoma (HCC) specimens that had been histopathologically and clinically diagnosed as LIHC were obtained from the Department of Hepatobiliary Surgery of the Third Affiliated Hospital of Sun Yat-sen University. The clinicopathologic characteristics of the 77 patients are summarized in **Table S1**. Furthermore, ten fresh HCC tissue samples, together with their paired adjacent non-cancerous tissues from each patient, were collected from HCC curative resection surgery. The Research Ethics Committee of the Third Affiliated Hospital was obtained for the use of the clinical materials described above for research purposes.

### Cell culture and transfection

Two HCC cell lines (HepG2, SNU-449) were obtained from the Key Laboratory of Liver Disease of Guangdong Province. The cells were cultured in Dulbecco’s Modification of Eagle’s Medium (DMEM, Gibco, Carlsbad, CA, USA) supplemented with 10% fetal bovine serum (FBS, Gibco) at a temperature of 37°C and 5% CO2. Regular digestion and passage of the cells were performed to maintain their growth and viability. Cells were transfected with siRNA using Lipofectamine 3000 (ThermoFisher) according to the manufacturer’s instructions. Cells were transiently transfected for 48 h for mRNA assessments. Sequences for siRNAs were as follows: siCtrl sense 5’-UUCUCCGAACGUGUCACGUTT-3’, antisense 5’-ACGUGACACGUUCGGAGAATT-3’; siG6PD-1 sense 5’-ACAGAUACAAGAACGUGAATT-3’, antisense 5’-UUCACGUUCUUGUAUCUGUUG-3’; and siG6PD-2 sense 5’-CGUCCUCUAUGUGGAGAAUTT-3’, antisense 5’-AUUCUCCACAUAGAGGACGAC-3’.

### Reverse transcription and quantitative PCR

Total RNA was isolated from tissue specimens and HCC cell lines using Trizol reagent (Invitrogen, Carlsbad, CA, USA) according to the manufacturer’s protocol. Reverse transcription was then performed to generate cDNA using a Reverse Transcription Kit (Takara, Cat: RR036A, KeyGEN). qPCR conditions were as follows: 10 min at 95°C followed by 35–40 cycles at 95°C for 15s and 60 °C for 34s, followed by a plate read after each cycle. Relative RNA levels were calculated using the comparative 2-ΔΔCT method with β-actin serving as an endogenous control. The primers were as follows: G6PD, forward: 5′-ATGCCTTCCATCAGTCGGAT-3′and reverse: 5′AGCCCACGATGAAGGTGTTT-3′; β-actin, forward: 5′- GCACCCAGCACAATGAAGAT-30 and reverse: 5′- ACATCTGCTGGAAGGTGGAC-3′.

### Cell proliferation assays

HepG2 cell lines and SNU-449 cell lines with or without G6PD silencing were seeded into 96-well plates (100 μl cell suspensions). Cell numbers were assessed every 24 h by CCK-8 assays according to the manufacturer’s instructions. The reaction products were measured at 450 nm.

### Statistical analysis

All gene expression data underwent transformative normalization using the log2 function. Student’s t-test was used to analyze the gene expression data sets from the TCGA and GTEx databases. Spearman’s Rank-Correlation test or Pearson correlation analysis was used to determine correlations between two variables. Both the Kaplan-Meier method and log-rank test were applied to calculate the HR and their P values to compare survival curves. Results with a P value of less than 0.05 were considered statistically significant.

## Results

### G6PD expression is dysregulated in human pan-cancer

**Figure 1:**
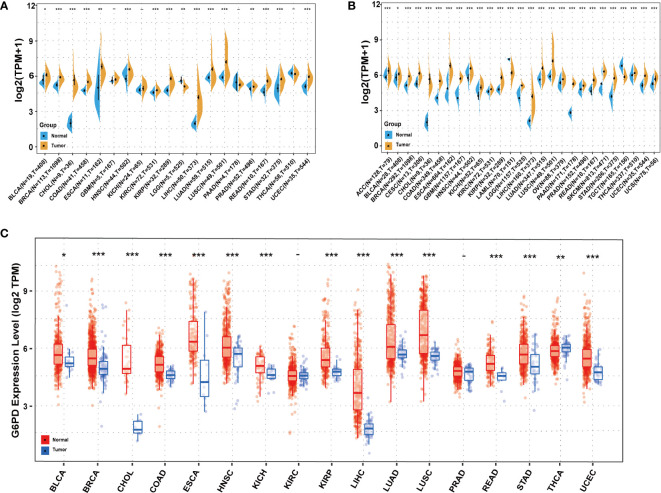
Expression levels of G6PD in different tissues in pan-cancer **(A)** Expression levels of G6PD in tumor and paired adjacent noncancerous tissues containing 20 tissues from TCGA. **(B)** G6PD expression difference in 27 tumors integrating data of normal tissues in GTEx database and tumor tissues in TCGA database. **(C)** Human G6PD expression levels in different cancer types from TCGA database in TIMER. *p <0.05, **p <0.01, ***p < 0.001.

Initially, to reveal G6PD expression specificity, we utilized the data from the TCGA database to analyze the differences in G6PD expression levels in tumor and adjacent normal tissues. The result demonstrated that, compared to normal tissues, there was a significant increase in G6PD expression levels in BRCA, CHOL, COAD, ESCA, HNSC, KIRC, KIRP, LIHC, LUAD, LUSC, READ, STAD, and UCEC (P<0.05) (**Figure 1A**). Due to the absence of normal tissue expression profiles in the TCGA database, we integrated the data from the TCGA database and the GTEx database to conduct a thorough analysis. The results revealed that G6PD expression levels were relatively high in 25 types of cancer, except TGCT and LAML (**Figure 1B**). Upon examining the TIMER database, it was discovered that the expression of G6PD was significantly elevated in various types of cancer, including BLCA, BRCA, CHOL, COAD, ESCA, HNSC, KICH, KIRP, LIHC, LUAD, LUSC, READ, STAD, and UCEC when compared to their normal counterparts. Conversely, the expression of G6PD was found to be lower in THCA (**Figure 1C**). Taken together, the above data suggested that G6PD was abnormally expressed in different cancers and it might play a critical role in tumor pathophysiology.

### Prognostic value of G6PD in pan-canceR

**Figure 2:**
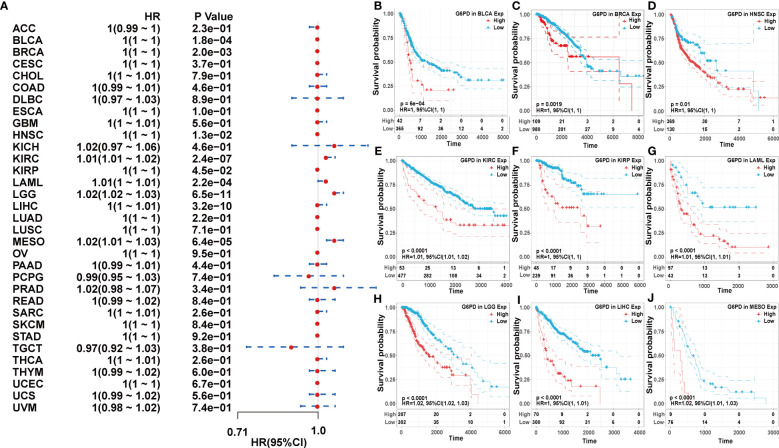
Association of G6PD expression with patient overall survival (OS). **(A)** The forest plot shows the relationship of G6PD expression with patient OS. **(B–J)** Kaplan-Meier analyses show the association between G6PD expression and OS.

Next, we used the one-way Cox regression model to elucidate the relationship between G6PD levels and OS of patients in the TCGA cohort. The forest plots across the 33 tumors demonstrated that high G6PD levels were associated with increased risk in KIRC, LAML, LGG, and MESO. Using the KM plotter portal and the log-rank method, we further evaluated the relationship between G6PD expression levels and patient outcomes. KM analysis revealed that patients with higher G6PD levels experienced shorter overall survival compared with patients with lower G6PD levels in BLCA, BRCA, HNSC, KIRP, LIHC, KIRC, LAML, LGG, MESO (**Figure 2**). Similarly, the association of G6PD expression with patients’ DSS was also investigated. One-way Cox regression analysis revealed that G6PD expression had a significant impact on the DSS of patients with KIRC, LGG, MESO, and PRAD. KM plotter portal indicated that high expression of G6PD was significantly associated with poor DSS for patients with KIRC, LGG, MESO, PRAD, and THCA (**Figure S1**). In addition, a forest plot indicated that G6PD expression significantly affected DFI in MESO, PRAD, and STAD. Moreover, KM curves also found that G6PD overexpression was associated with poor DFI in MESO, PRAD, and STAD. However, increased levels of G6PD indicated better DFI in OV (**Figure S2**). Meanwhile, KM curves confirmed that patients with high G6PD expression had a worse PFI than those with lower G6PD expression in many cancers, such as KIRC, LGG, MESO, and PRAD. And the one-way Cox regression analysis of PFI revealed that higher G6PD expression indicated a risk factor in COAD, KIRC, LGG, MESO, and PRAD (**Figure S3**). The results above suggested a negative correlation between G6PD expression and survival time in some types of cancer. Overall, these data showed that G6PD might be a prognostic biomarker associated with patient OS, DSS, DFI, and PFI in various human cancers, especially in LIHC.

### Correlation between G6PD and clinical characteristics

**Figure 3:**
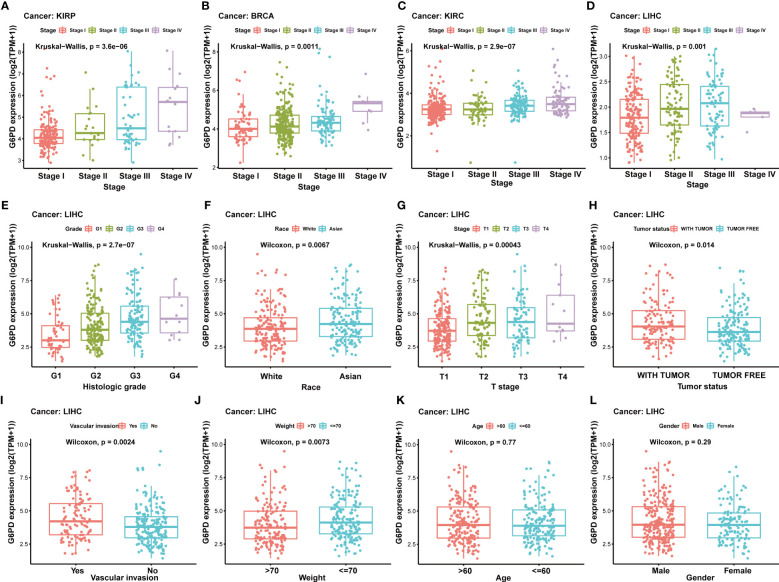
Correlation between G6PD and clinical characteristics **(A–F)** Correlation between G6PD expression and pathological stages of KIRP, BRCA, KIRC, and LIHC from TCGA datasets. **(G–L)** The correlation between G6PD expression and Clinical Characteristics in LIHC, including the histologic stage, tumor status, pathologic stage, TNM-T stage, vascular invasion, race, BMI, gender, age and weight. Log2 (TPM + 1) was applied for the log scale.

Due to a limited understanding of the clinical value of G6PD expression in pan-cancer prognosis, we conducted further analysis to detect the association of G6PD expression with clinicopathological characteristics in nine cancers including BLCA, BRCA, HNSC, KIRP, LIHC, KIRC, LAML, LGG, MESO, all of which exhibited significant prognostic value, as determined by the aforementioned univariate Cox analysis. As shown in **Figure 3**, there was a positive correlation between G6PD expression levels and tumor stages, such as KIRP, BRCA, KIRC, and LIHC. The results of the analysis suggest a potential association between the expression level of G6PD and the prognosis of patients. Additionally, we further evaluated the association between G6PD expression and clinical features in LIHC, which revealed that elevated G6PD expression was strongly correlated with tumor status, vascular invasion, risk factor, T stage of the TNM classification, histologic grade, race, and weight (**Figures 3E–L**), confirming the positive correlation between G6PD expression and LIHC progression.

### Pan-cancer association analysis of G6PD expression and tumor immune infiltration

**Figure 4:**
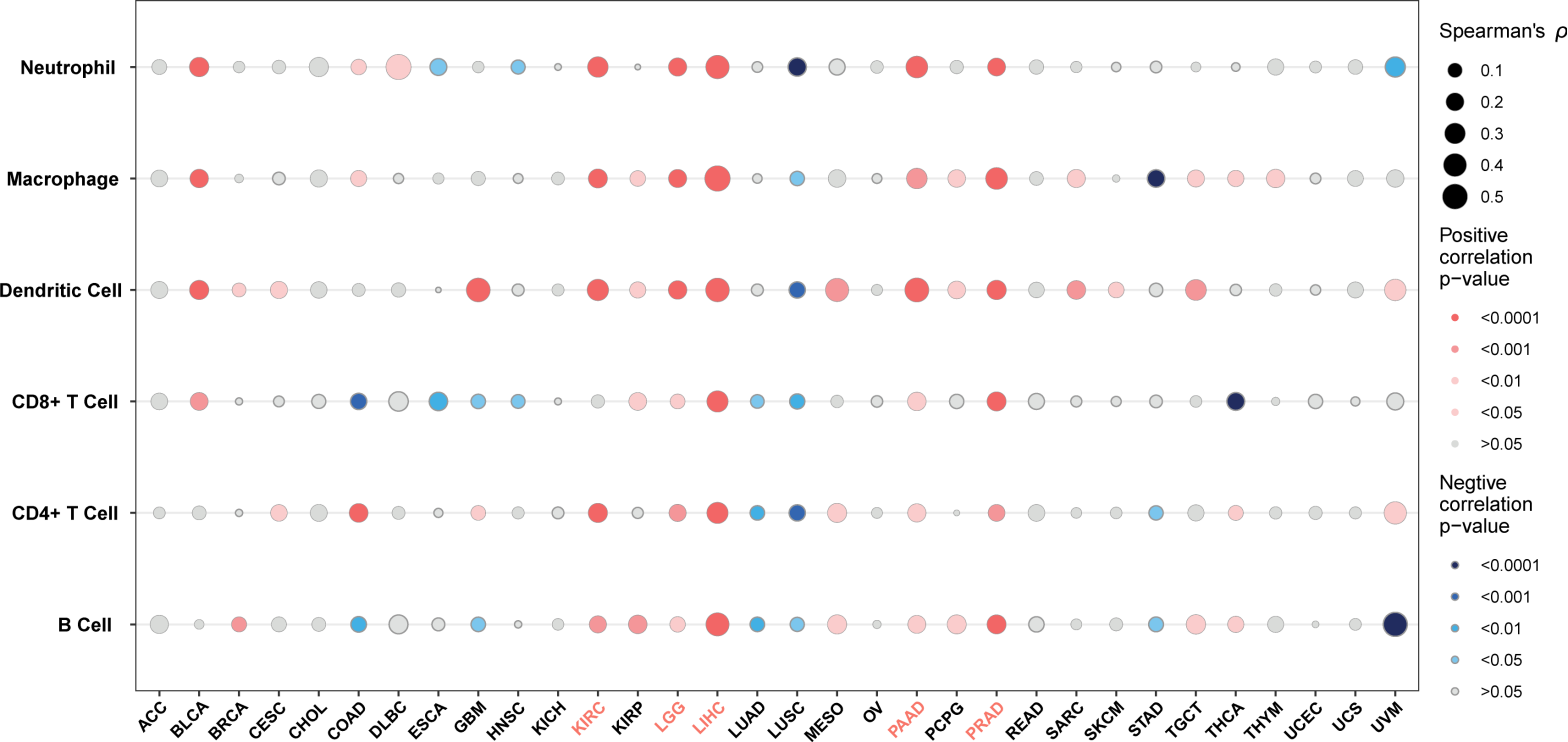
Correlation between G6PD expression levels and immune cell infiltration.

**Figure 5:**
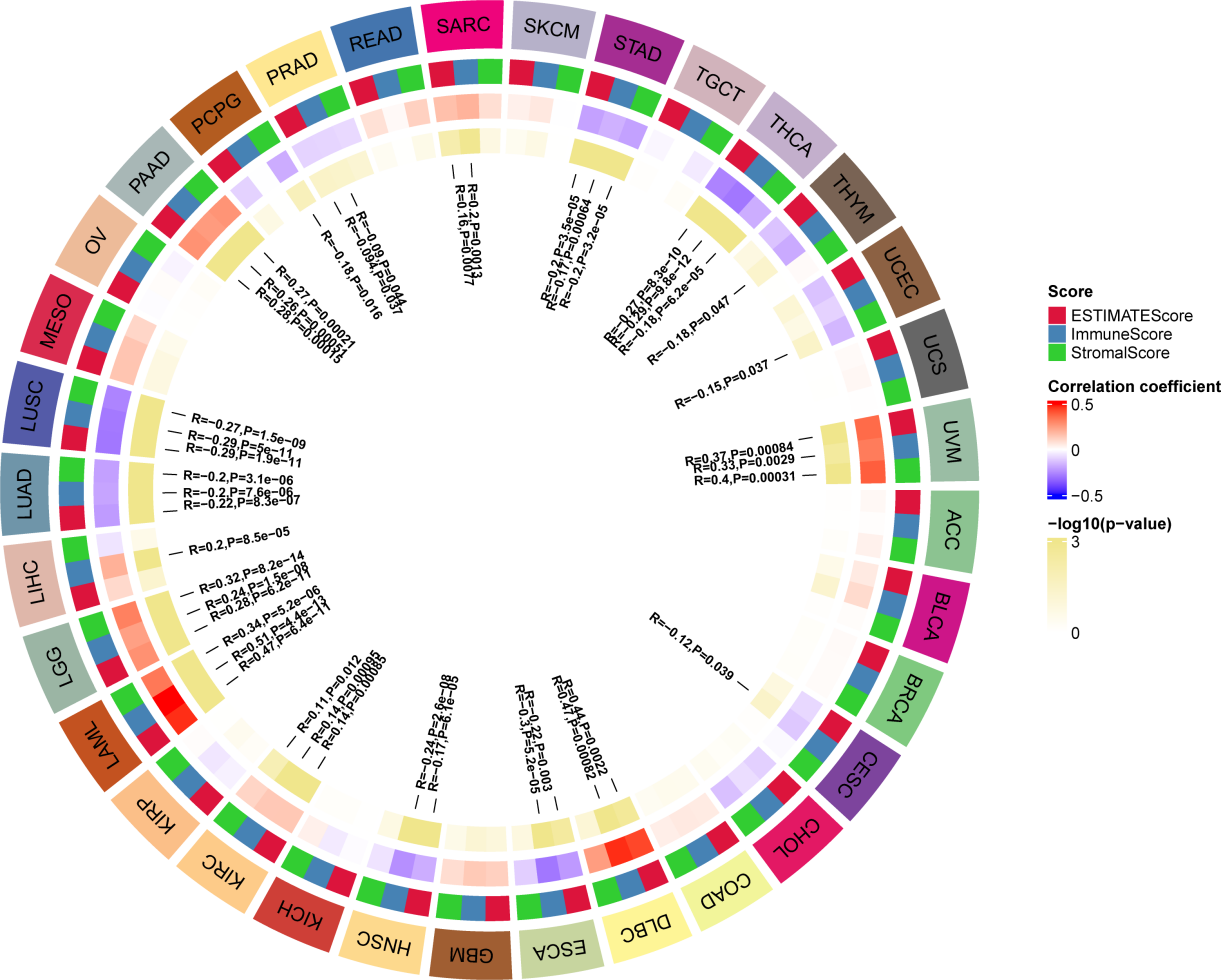
Correlation of G6PD with the immune score, stromal score, and ESTIMATE score in pan-cancer.

TME contains an extracellular matrix, relevant factors, and diverse infiltrating immune cells, such as regulatory T cells, B cells, neutrophils, and macrophages, as well as natural killer cells and dendritic cells. Thus, we evaluated the relationship between G6PD expression and levels of immune infiltration across cancers. we observed that there was an association between G6PD expression and six infiltrating immune cells in a majority of tumors, and the top five cancers were KIRC, LGG, LIHC, PAAD, and PRAD (**Figure 4**). Next, to quantify the immune and matrix components in pan-cancer, we calculated the Immune Score, Stromal Score, and ESTIMATES Score in tumor samples by using the estimate algorithm in R software (24). As shown in **Figure 5**, the top three cancers were UVM (R =0.4, P <0.001), LAML (R =0.34, P <0.001), and LGG (R = 0.32, P <0.001), in which G6PD expression was most closely associated with the Stromal Score. Moreover, the first three cancers with G6PD expression closely linked to Immune Score were LAML (R =0.51, P <0.001), DLBC (P=0.47, P <0.001), and UVM (R =0.33, P <0.001). As for ESTIMATEScore, the first three cancers were LAML (R = 0.47, P <0.001), DLBC (= 0.44, P<0.05), and UVM (R =0.37, P <0.001). In addition, the Stromal Score, immune Score, and ESTIMATEScore of LUAD, LUSC, PRAD, STAD, and THCA were negatively associated with G6PD expression. Taken together, these results revealed that G6PD might be involved in tumor immune response.

### Gene set enrichment analyses of G6PD

**Figure 6:**
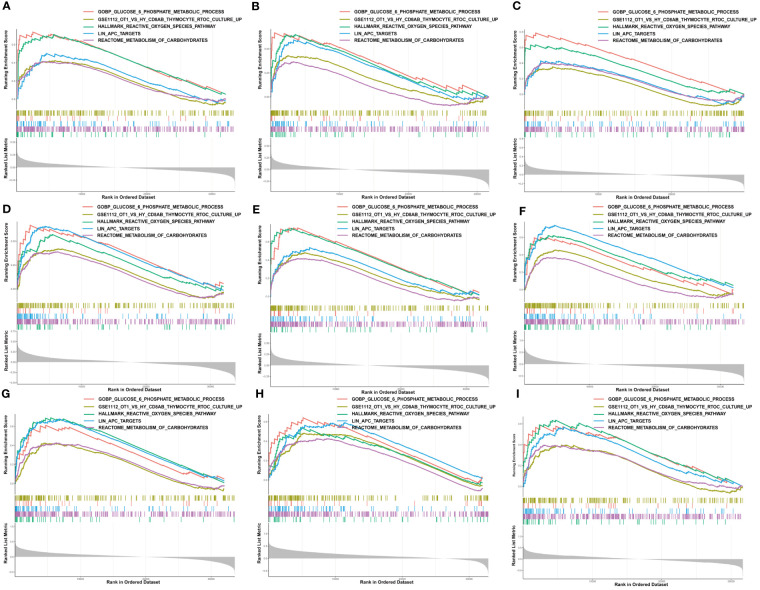
Gene set enrichment analysis of G6PD. The five common signaling pathways in nine tumors with prognostic significance. **(A)** Bladder urothelial carcinoma (BLCA); **(B)** Breast invasive carcinoma (BRCA); **(C)** Head and neck squamous cell carcinoma (HNSC); **(D)** Kidney renal clear cell carcinoma (KIRC); **(E)** Kidney renal papillary cell carcinoma (KIRP); **(F)** Acute myeloid leukemia (LAML); **(G)** Brain lower grade glioma (LGG); **(H)** Liver hepatocellular carcinoma (LIHC); **(I)** Mesothelioma (MESO).

Moreover, we performed GSEA to explore the common signaling pathways in the aforementioned nine tumors with prognostic significance. The result showed that a total of thirteen gene sets were commonly enriched in the nine prognostic tumors including BLCA, BRCA, HNSC, KIRC, KIRP, LAML, LGG, LIHC, and MESO. The five most common signaling pathways in the nine tumors were listed in **Figures 6A–I**, and the detailed enrichment results of LIHC were shown in **Table 1**. The GSEA results showed that the expression of G6PD was associated with metabolic-related activities, including ROS metabolism, carbohydrates, and glucose-6-phosphate on the one hand, and tumor immune responses including humoral immunity and cellular immunity on the other hand. Meanwhile, we also found that the differentially expressed genes (DEGs) were enriched in the LIN_APC_TARGETS gene set. The complete GSEA results were shown in **Table S2**. These results gave insight into the role of G6PD in cancer establishment and development.

**Table 1:** The information of GSEA enrichment analysis of LIHC.

gene sets	ES	NES	NP	FDR	FWER
**HALLMARK_UV_RESPONSE_UP**	0.4472	1.7984	0	0.0304	0.101
**HALLMARK_REACTIVE_OXYGEN_SPECIES_PATHWAY**	0.4751	1.6558	0.0269	0.0623	0.285
**REACTOME_METABOLISM_OF_CARBOHYDRATES**	0.4244	1.7247	0	0.0743	0.911
**LIN_APC_TARGETS**	0.4964	1.7431	0.0084	0.0714	0.879
**GOBP_GLUCOSE_6_PHOSPHATE_METABOLIC_PROCESS**	0.5499	1.6974	0.0105	0.1061	0.956
**HP_MIDDLE_AGE_ONSET**	0.4676	1.6221	0.0143	0.094	0.986
**NAIVE_VS_MEMORY_BCELL_DN**	0.4632	1.8276	0	0.0035	0.19
**OT1_VS_HY_CD8AB_THYMOCYTE_RTOC_CULTURE_UP**	0.4734	1.8464	0	0.0032	0.158
**WT_VS_CTLA4_KO_CD4_TCELL_D4_POST_IMMUNIZATION_DN**	0	0.0019	0.068	0	0.0019
**BTLA_POS_VS_NEG_INTRATUMORAL_CD8_TCELL_UP**	0.5348	2.0077	0	0.0019	0.025
**NAIVE_VS_IGM_MEMORY_BCELL_DN**	0.4504	1.7754	0.0021	0.005	0.276
**HOEK_NEUTROPHIL_2011_2012_TIV_ADULT_1DY_DN**	0.4823	1.7173	0.0039	0.0076	0.378
**UNTREATED_VS_6H_NOD2_LIGAND_TREATED_MONOCYTE_UP**	0.4482	1.6913	0.0041	0.0092	0.423

ES, Enrichment score; NES, standardized enrichment score; NP, nominal p-value; FDR, false discovery rate; FWER, Family-wise error rate.

### Analysis of G6PD drug resistance and molecular docking

**Figure 7:**
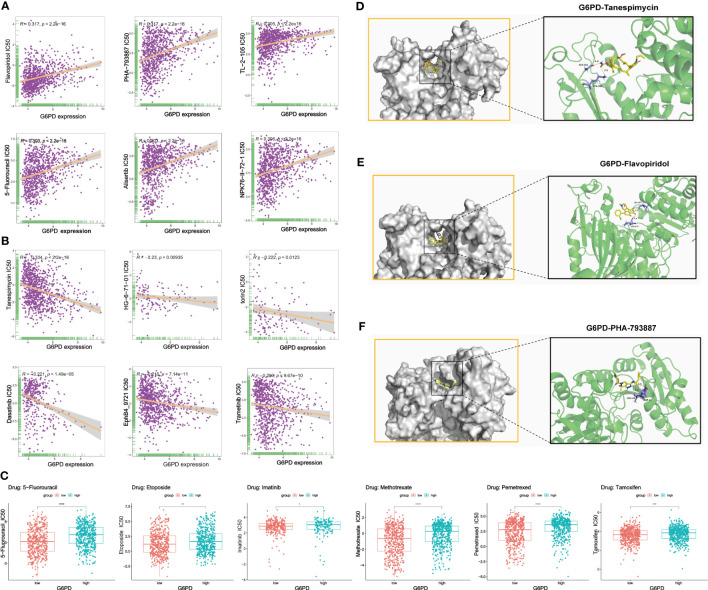
Correction between G6PD expression and drug sensitivity. **(A)** The top six positively correlated. **(B)** The top six negatively correlated. **(C)** The difference in drug sensitivity of six commonly used anti-cancer drugs (5-fluorouracil, Etoposide, Imatinib, Methotrexate, and Tamoxifen) in high and low G6PD expression levels. Molecular docking results of protein G6PD (2BHL) with Tanespimycin **(D)**, Flavopiridol **(E)**, and PHA-793887 **(F)**. There are two hydrogen bonds of 1.9, and 2.5 Å in the docking between G6PD and Tanespimycin. The docking results of G6PD and Flavopiridol showed two hydrogen bonds of 2.1 Å and 2.6 Å respectively. Two hydrogen bonds of 2.3 and 2.6Å were found between G6PD and PHA-793887. *p < 0.05; **p < 0.01; ***p < 0.001; ****p < 0.0001.

Genetic alterations can affect susceptibility to antitumor drugs and previous studies have revealed that G6PD is implicated in resistance to some chemotherapy drugs (30, 31). Therefore, we performed the analysis of G6PD drug resistance based on the GDSC database and identified 252 drugs (403 in total) that were significantly associated with G6PD expression in 970 cancer cell lines. Among them, 84% of the identified drugs’ IC50s were positively correlated with G6PD expression. The top six drugs with the strongest positive and negative correlation between IC50s and G6PD expression were shown in **Figures 7A, B** respectively. Detailed drug sensitivity analysis data were shown in **Table S3**. Meanwhile, six commonly used anti-cancer drugs including 5-fluorouracil, Etoposide, Imatinib, Methotrexate, and Tamoxifen had higher IC50 values in patients with higher G6PD expression levels (**Figure 7C**). These results revealed that the G6PD expression level may be related to the sensitivity of chemotherapy drugs and might provide a new pathway for clinical cancer treatment. Further, given the strong correlation existing between G6PD expression levels and chemotherapy drug sensitivity, molecular docking was conducted to determine the potential binding abilities of these drugs with G6PD. The results demonstrated that the binding free energy of G6PD protein to Tanespimycin, Flavopiridol, and PHA-793887 was -5.61, -5.77, and -6.18 kcal/mol respectively. Additionally, there were two hydrogen bonding forces between the G6PD protein and the three drugs (**Figures 7D–F**). Taken together, the results above suggested that various chemotherapeutic drugs were showing excellent binding activity with G6PD.

### Protein-protein interactions network

**Figure 8:**
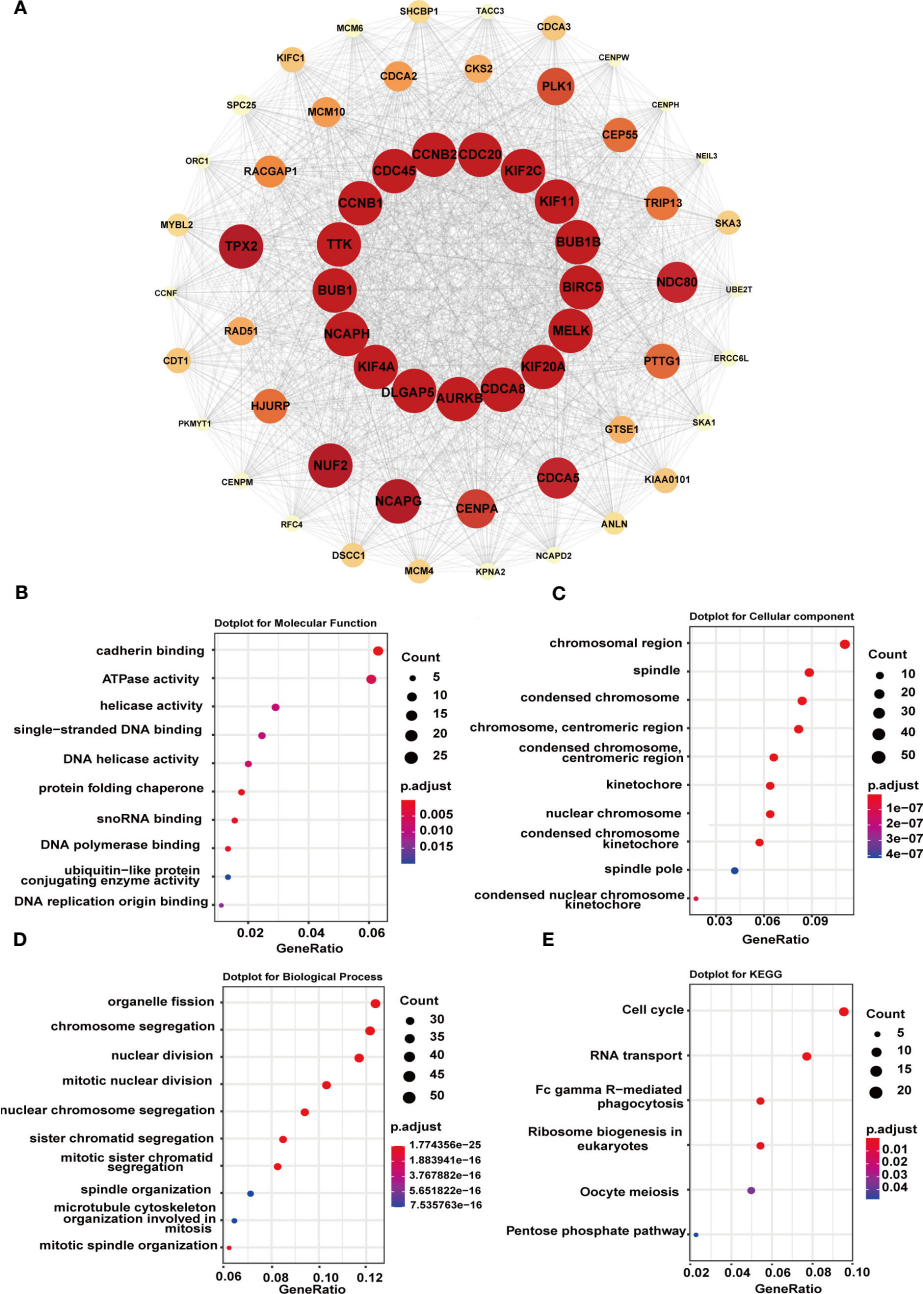
Functional enrichment analysis of G6PD-related genes. **(A)** STRING protein network map of top 60 experimentally determined G6PD-binding proteins. The color, shape, and font size represent the strength of the relationship. **(B–D)** The top 10 GO enrichment significance terms of G6PD-related genes of LIHC in three functional groups: molecular function (MF), cell component (CC), and biological process (BP). **(E)** KEGG pathway enrichment analyses of the G6PD correlation network of LIHC.

Next, to figure out the potential mechanism of G6PD’s roles in tumorigenesis and tumor progression of LIHC, we constructed a PPI network including 1566 edges and 60 nodes. Then, based on the MCODE plugin in Cytoscape, the hub genes were identified (MCODE Score=53.085). As shown in **Figure 8A**, there were seventeen top hub genes, and most of them are involved in DNA replication, mitosis, and regulation of the cell cycle, such as CDC20, KIF2C, BUB1, AURKB, and NCAPH. To further investigate the possible biological functions of G6PD, we performed GO term and KEGG pathway analysis in LIHC. The KEGG pathway analysis result indicated that G6PD PPI was primarily enriched in the cell cycle and RNA transport pathway, which is closely related to the cell proliferation process (**Figure 8E**). Similar to KEGG, the GO term analysis was intimately involved in “cadherin binding and ATPase activity” in the molecular function (MF) category, “chromosomal region and spindle” in the cellular component (CC) category and “organelle fission and chromosome segregation” in biological process (BP) category (**Figures 8B–D**). The results above imply that G6PD PPI might contribute to cancer progression by regulating signaling pathways related to cell proliferation.

### The functional validation of G6PD

**Figure 9:**
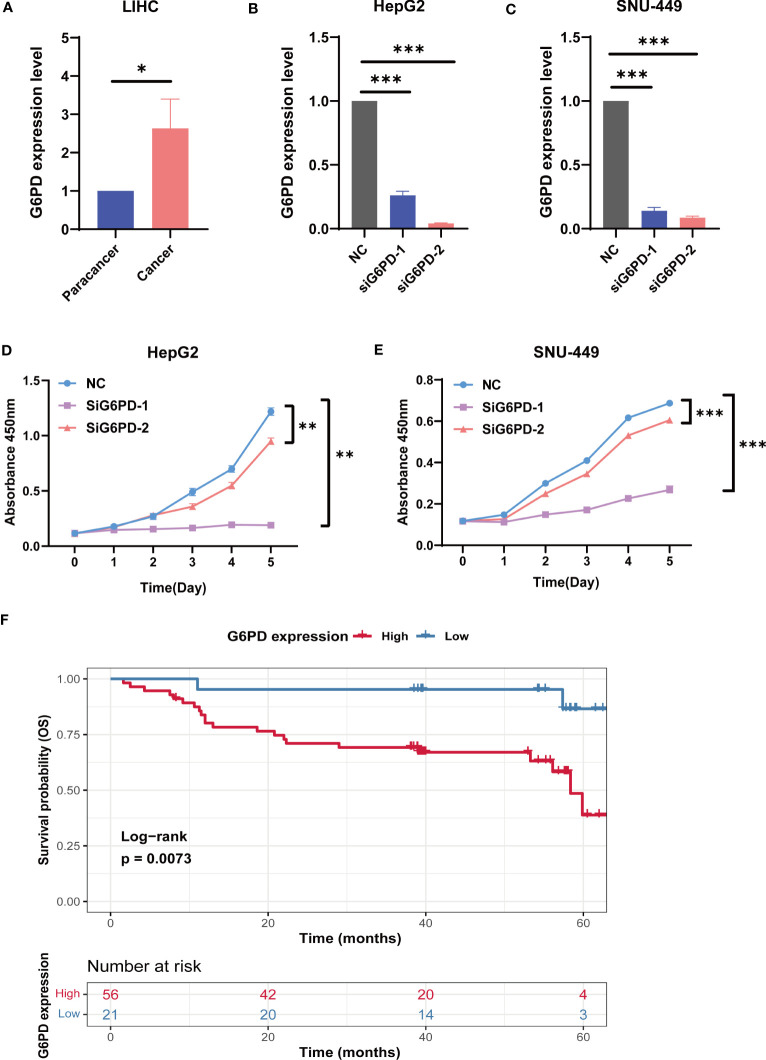
The biological functions of G6PD in LIHC. **(A)** The expression level of G6PD in 10 fresh HCC specimens and corresponding adjacent normal tissues. **(B, C)** Verification of knockdown efficiency of G6PD in HepG2 cell lines and SNU-449 cell lines. **(D, E)** The biological functions of G6PD on HCC cell lines were verified by CCK-8 assays. **(F)** Association between G6PD expression and overall survival (OS) time in patients with HCC. (Tumor specimens were from the Department of Hepatobiliary Surgery of the Third Affiliated Hospital of Sun Yat-sen University). *p <0.05, **p <0.01, ***p < 0.001.

Next, we analyzed G6PD expression levels in HCC specimens and HCC cell lines using qRT-PCR. We examined the expression level of G6PD in 10 fresh HCC samples and corresponding adjacent normal tissues. We found that the expression of G6PD in tumor tissues was significantly higher than that in the corresponding adjacent normal tissues, which was consistent with the results of the TCGA database (**Figure 9A**). We then used specific G6PD-targeting siRNAs to knock down the expression levels of G6PD in HepG2 cell lines and SNU-449 cell lines (**Figures 9B, C**). The CCK-8 assay showed that the cell viability of the G6PD knockdown group was markedly decreased compared to that of the negative control (NC) group (**Figures 9D, E**). Furthermore, a survival analysis involving 77 patients with HCC from the Third Hospital of Sun Yat-sen University unveiled a worse prognosis and shorter OS in patients with high G6PD expression (**Figure 9F**). These compelling results imply a potential association between G6PD and hepatocellular carcinoma cell proliferation, as well as an unfavorable prognosis in patients afflicted with HCC.

## Discussion

A preponderance of research has revealed that G6PD is involved in energy balance, especially in blood-related diseases (32). Furthermore, studies have shown that G6PD is intimately related to the occurrence and development of tumors and critical for signaling pathways that control cell proliferation and cell death in rapidly growing cancer cells (33). However, most of the previous studies focused only on a single cancer type or disease. The prognostic value, tumor immunity, and biological significance of G6PD have not been well understood. Unfortunately, there is a lack of pan-cancer analysis of G6PD. Hence, we comprehensively analyzed the G6PD gene in various tumors using public databases to explore the landscape of G6PD expression, gene functional enrichment analysis, immune microenvironment, prognostic value, and drug resistance.

According to our investigations, upregulated expression of G6PD has been identified in many tumor cells and elevated G6PD expression levels are indicative of unfavorable clinical outcomes in cancer patients (34). For instance, G6PD is highly expressed in HCC and has been shown to contribute to the metastasis and poor prognosis of HCC (35). Besides, G6PD was significantly upregulated in lung adenocarcinoma (LUAD) cell lines and promoted proliferation and migration *in vivo* (36). Similar to previous studies, our results showed that G6PD is significantly upregulated in the overwhelming majority of tumors compared to their corresponding adjacent normal tissues (**Figure 1**). However, the level of G6PD expression in cancer tissues of TGCT was significantly lower compared to the corresponding normal tissues, which was generally counterintuitive since G6PD was highly expressed in testicular tissues (**Figure 1B**). Notably, the upregulated expression of G6PD correlated with worse prognosis in several cancers, including BLCA, BRCA, HNSC, KIRP, LIHC, KIRC, LGG, MESO, and LAML (**Figure 2**). In the wake of further analysis of the clinical data of tumor patients from TCGA, we found the G6PD expression was positively correlated with the tumor stage in various cancers, and higher G6PD expression was significantly associated with vascular invasion, TNM-T stage, histologic grade, tumor status, weight, and race in LIHC (**Figure 3**). Supporting that, G6PD was found to be involved in the regulation of cell proliferation, angiogenesis, distant metastasis, and chemotherapy drug resistance in HCC, BRCA, and KIRC (14, 35, 37). Together, these results suggested that high G6PD expression was a risk factor in various cancers and closely related to tumor progression.

Numerous studies have explored the molecular mechanisms and possible signaling pathways of G6PD involved in tumorigenesis. Combined with previous studies, we believe that the regulation of G6PD on tumor cells and its impact on prognosis is multifaceted. We speculated that the possible mechanisms are involved in metabolic reprogramming, regulating cell proliferation and apoptosis, and participating in tumor immunity and anti-tumor drug resistance.

The metabolic reprogramming and the imbalance of energy metabolism in tumor cells have been the core of existing studies (38). Unlimited proliferation, angiogenesis, and genomic instability are the most typical characteristics of tumor cells. To match the increased proliferation rate and nutrient consumption, tumor cells undergo many metabolic changes. Even under oxygen-rich conditions, tumor cells metabolize glucose to lactate instead of entering the tricarboxylic acid cycle to support their rapid growth and proliferation, known as the “Warburg effect” or “aerobic glycolysis” (39). As a key rate-limiting enzyme of PPP, G6PD plays an important role in this metabolic reprogramming of tumors (40). PPP supplies NADPH and produces large amounts of ribose 5-phosphate (R-5-P), both of which are closely related to cell proliferation (17). In line with this, our GSEA results suggested that G6PD was significantly involved in the metabolic-related signaling pathways including active oxygen metabolism pathway, carbohydrate and glucose 6-phosphate metabolism pathway. By controlling GSH, which is used to detoxify high levels of reactive oxygen species (ROS) produced during rapid cellular proliferation, NADPH can increase cellular antioxidant capacity and aid in cell survival (41). NADPH/NADP^+^ ratio can regulate the G6PD enzyme activity and PPP flux (17). In addition, pentose required by tumor cells for over 85% nucleotide synthesis is directly or indirectly provided through PPP (33). Based on the above, it is not surprising that G6PD can affect the development of tumors, and interfering with ROS status in tumor cells may be a meaningful therapeutic direction.

In addition to tumor metabolism, G6PD can widely affect tumor cell proliferation and apoptosis. ROS plays a key role in regulating the survival of tumor cells, which is a double-edged sword. On the one hand, ROS can activate the cell mitotic signaling pathway to promote cell proliferation and angiogenesis. And on the other hand, ROS can make cells sensitive to apoptotic signals and accelerate the process of cell apoptosis (42, 43). Evidence has shown that blocking G6PD activity can reduce cancer cell proliferation and increase apoptosis (44). Oncogenes and suppressor genes can respectively increase or suppress G6PD expression, such as Myc and P53 (44, 45). For example, Zhang et al. have found that G6PD facilitates renal cell carcinoma proliferation through a positive feedback loop involving the activation of the G6PD/ROS/p−STAT3/Cyclin D1 axis (9). Our GSEA results also found that the DEGs were involved in the LIN_APC_TARGETS genset (**Figure 6**). Strikingly, in agreement with previous studies showing that G6PD is related to cell cycle proteins (46), our PPI network results also showed that almost all the top hub genes were involved in DNA replication, mitosis and regulation of the cell cycle, such as CDC20, KIF2C, BUB1, AURKB, NCAPH (**Figure 8A**). Among them, one of the hub genes was also associated with the inhibition of apoptosis. Conforming to the PPI network, the KEGG pathway enrichment analysis confirmed that G6PD was involved in the cell cycle and RNA transport pathway (**Figure 6E**). Meanwhile, GO analysis also found that G6PD was involved in the chromosomal region and spindle in the cellular component category (**Figure 8C**). Therefore, it is reasonable to believe that the G6PD protein may have other biological functions in regulating tumor cell growth besides the enzyme activity of the PPP pathway. Studies have found that G6PD can sufficiently support AMPK activation independent of its enzymatic activity (47). The detailed biological function of G6PD in tumor cells still needs to be studied.

Another key finding of our study is that the G6PD expression was highly associated with immune infiltration. Tumor-infiltrating lymphocytes (TILs; including B cells and T cells) and other immune cells (dendritic cells, neutrophils, and macrophages) are essential components of the TME. TILs have been demonstrated to be a reliable predictor of prognosis (48). Remarkably, based on the results of this study, we found that G6PD expression was significantly positively correlated with infiltrating immune cells in most cancer types, especially in KIRC, LGG, LIHC, and PAAD, while negatively correlated with LUSC, STAD (**Figure 4**). Next, based on the method of ESTIMATE, we calculated the Immune, Stromal, and ESTIMATE scores. Our analysis showed that G6PD expression was positively correlated with the Immune Score and Stromal Score in some cancers, especially in LAML, LGG, DLBC, and PAAD (**Figure 5**). A higher Immune Score or Stromal Score in the TME denotes the presence of more immune or matrix components. Moreover, Our GSEA results also found that the DEGs were involved in tumor immune response, including cellular and humoral immunity (**Table 1**). This suggests that G6PD may represent a promising therapeutic target for immunotherapy in the treatment of these types of cancers.

Although immune invasion has different or even opposite effects in different tumor tissues. It is reasonable to assume that G6PD can affect the immune infiltration pathway and play a significant role in the occurrence and progression of tumors. The overlapping metabolic reprogramming in both cancer and immune cells has been widely explored (49). Jonathan et al. found that G6PD plays a role in stimulating the metabolism of CD4^+^T cells and CD8^+^T cells, and the inhibitor of G6PD can raise the NADP^+^/NADPH ratio and eventually suppress both their proliferative ability and immunological response (50). Therefore, the pattern of tissue metabolism is closely related to the immune response and can impact the differentiation and effector function of immune cells (51). Recent studies have found that there is metabolic competition between immune cells and tumor cells, and tumor metabolites have extensive effects on immune cells, which can mediate tumor immune evasion (52, 53). These findings support our results and clarify the immunological roles of G6PD in cancer cells. Therefore, metabolic intervention could provide an effective approach to cancer treatment.

Moreover, apart from involvement in metabolism, cell proliferation and apoptosis, and tumor immunity, the high expression of G6PD plays a pivotal role in chemotherapy resistance (31). In this study, we found that the IC50 value of most identified anti-cancer drugs was positively correlated with the G6PD expression level. In other words, high expression of G6PD is indeed involved in anti-tumor drug resistance. The specific mechanism is still unclear, which may be related to the redox environment in tumor cells (54). To date, anti-tumor drugs inhibiting G6PD or PPP have been widely applied or are undergoing clinical trials, such as Polydatin, Dehydroepiandrosterone (DHEA) (55). However, their non-specific effect target and extensive adverse reactions often limit their clinical application and further research is needed. In our study, we found that the G6PD protein exhibited excellent binding activity with various drugs, such as Tanespimycin, Flavopiridol, and PHA-793887. Although these observations still need confirmation by experimental approaches, targeted therapy for G6PD is possible.

Finally, even though many efforts have been made in investigating the information from different databases, there are some limitations in our study. Firstly, this study is based on public databases. The results need to be further confirmed by combining *in vitro* and *in vivo* experiments and clinical studies. The mechanism of G6PD in different cancer types needs to be further elucidated on the cellular and molecular levels. Our next step is to experimentally confirm and clarify the role of G6PD in various types of cancer. In addition, there are relatively limited data for some rare tumors, increasing the possibility of false positives. Finally, epigenetics and post-translational modifications are of great value in regulating the activity of intracellular signaling pathways.

## Conclusion

In summary, the results of the present study indicated that the G6PD expression levels are higher in pan-cancer tissues relative to the expression level in normal tissues. High G6PD expression levels are correlated with poorer clinical prognosis in several cancers. And G6PD expression is increased in the infiltration levels of B cells, CD8^+^ T cells, CD4^+^ T cells, macrophages, neutrophils, and dendritic cells in many cancers, especially in KIRC, LGG, and LIHC. Meanwhile, Functional analysis and PPI network results revealed that G6PD was involved in metabolic-related activities, immune responses, cell proliferation, and apoptosis. Additionally, analysis of drug sensitivity showed that high expression of G6PD was involved in anti-tumor drug resistance. Therefore, G6PD may play a vital role in immune infiltration and may be a potential therapeutic target for tumor immunotherapy.

## Data availability statement

The original contributions presented in the study are included in the article/**Supplementary Material**. Further inquiries can be directed to the corresponding authors.

## Author contributions

The concept for the project was developed by XL. XL, JG, and YC designed this study and revised the manuscript. JP, HW, XC, YL, and XX collected data from the public database and performed the analysis of the clinical data of the project. JG, TZ, and BL analyzed and interpreted the other data. TZ and XS wrote the manuscript. All authors contributed to the article and approved the submitted version.

## Glossary

**Table:**

ACC	Adrenocortical carcinoma
BLCA	Bladder urothelial carcinoma
BRCA	Breast invasive carcinoma
CESC	Cervical squamous cell carcinoma
CHOL	Cholangiocarcinoma
COAD	Colon adenocarcinoma
DEGs	Differentially expressed genes
DFI	Disease-free interval
DLBC	Lymphoid neoplasm diffuse large B-cell lymphoma
DSS	Disease-specific survival
G6PD	Glucose-6-phosphate dehydrogenase
ESCA	Esophageal carcinoma
GBM	Glioblastoma multiforme
GSEA	Gene set enrichment analysis
GTEx	Genotype-tissue expression
HNSC	Head and neck squamous cell carcinoma
KEGG	Kyoto Encyclopedia of Genes and Genomes
KICH	Kidney chromophobe
KIRC	Kidney renal clear cell carcinoma
KIRP	Kidney renal papillary cell carcinoma
KM	Kaplan-Meier
IC50	half-maximal inhibitory concentration
LAML	Acute myeloid leukemia
LGG	Brain lower grade glioma
LIHC	Liver hepatocellular carcinoma
LUAD	Lung adenocarcinoma
LUSC	Lung squamous cell carcinoma
MsigDB	Molecular signatures database
NADPH	Nicotinamide adenine dinucleotide phosphate
NES	Net enrichment score
ROS	Reactive oxygen species
OS	Overall survival
PCPG	Pheochromocytoma and paraganglioma
PRAD	Prostate adenocarcinoma
PFI	Progression-free interval
PPP	Pentose phosphate pathway
READ	Rectum adenocarcinoma
SARC	Sarcoma
SKCM	Skin cutaneous melanoma
STAD	Stomach adenocarcinoma
TGCT	Testicular germ cell tumors
TAMs	Tumor-associated macrophages
TCGA	The cancer genome atlas
THCA	Thyroid carcinoma
THYM	Thymoma
TILs	Tumor-infiltrating lymphocytes
TIMER	Tumor immunity estimation resource
TME	Tumor microenvironment
UCEC	Uterine corpus endometrial carcinoma
UCS	Uterine carcinosarcoma
UCSC	University of California Santa Cruz
UVM	Uveal melanoma
