# Supplementary figures and images for: Pan-cancer analysis reveals that G6PD is a prognostic biomarker and therapeutic target for a variety of cancers
Source: Front Oncol. 2023 Aug 3;13:1183474. doi: 10.3389/fonc.2023.1183474 (PMC10435888; doi:10.3389/fonc.2023.1183474)

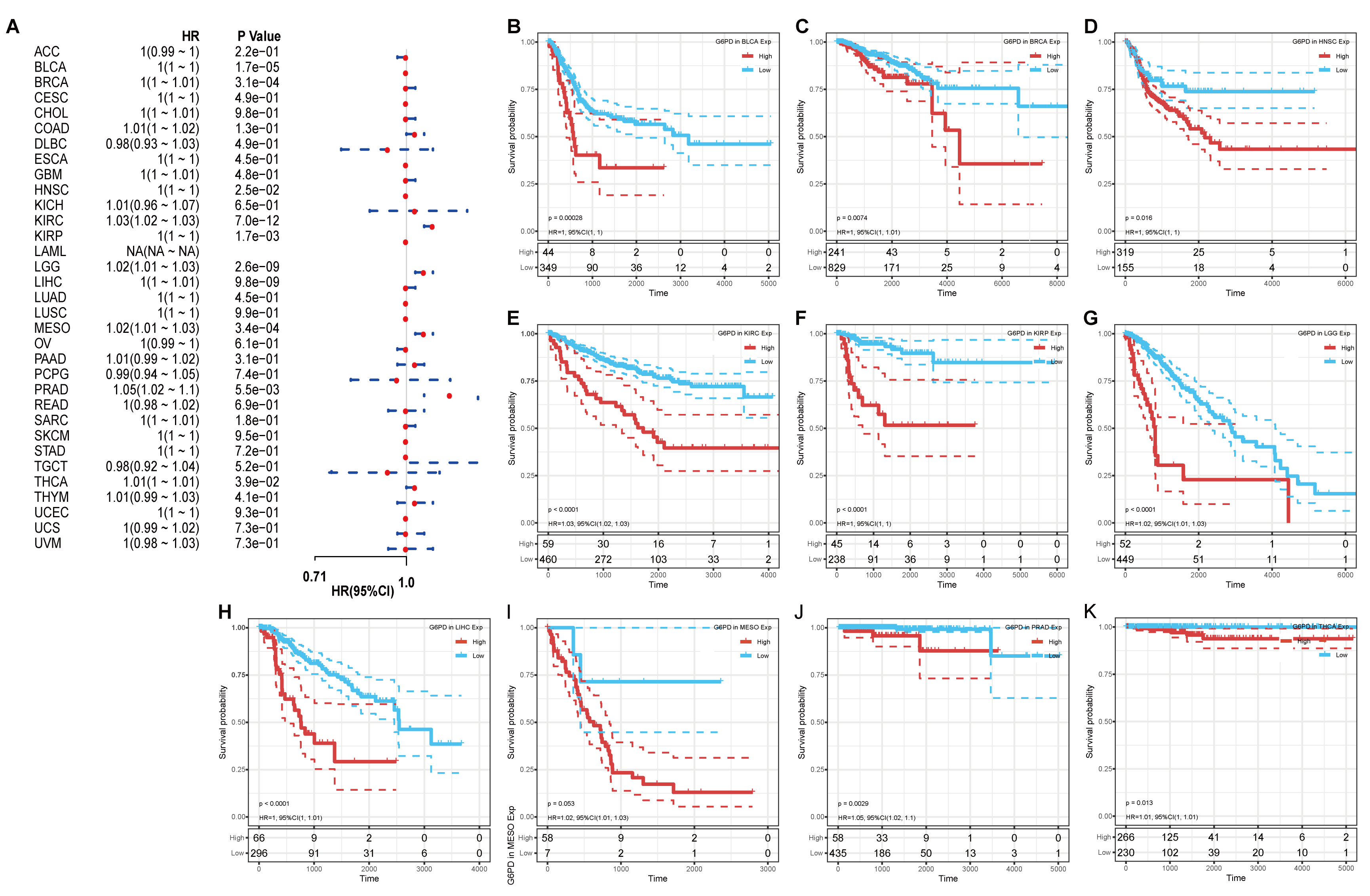

Supplement: Supplementary file 1 [file DataSheet_1.zip › Additional file/Additional file1/Figure S1 .tif]

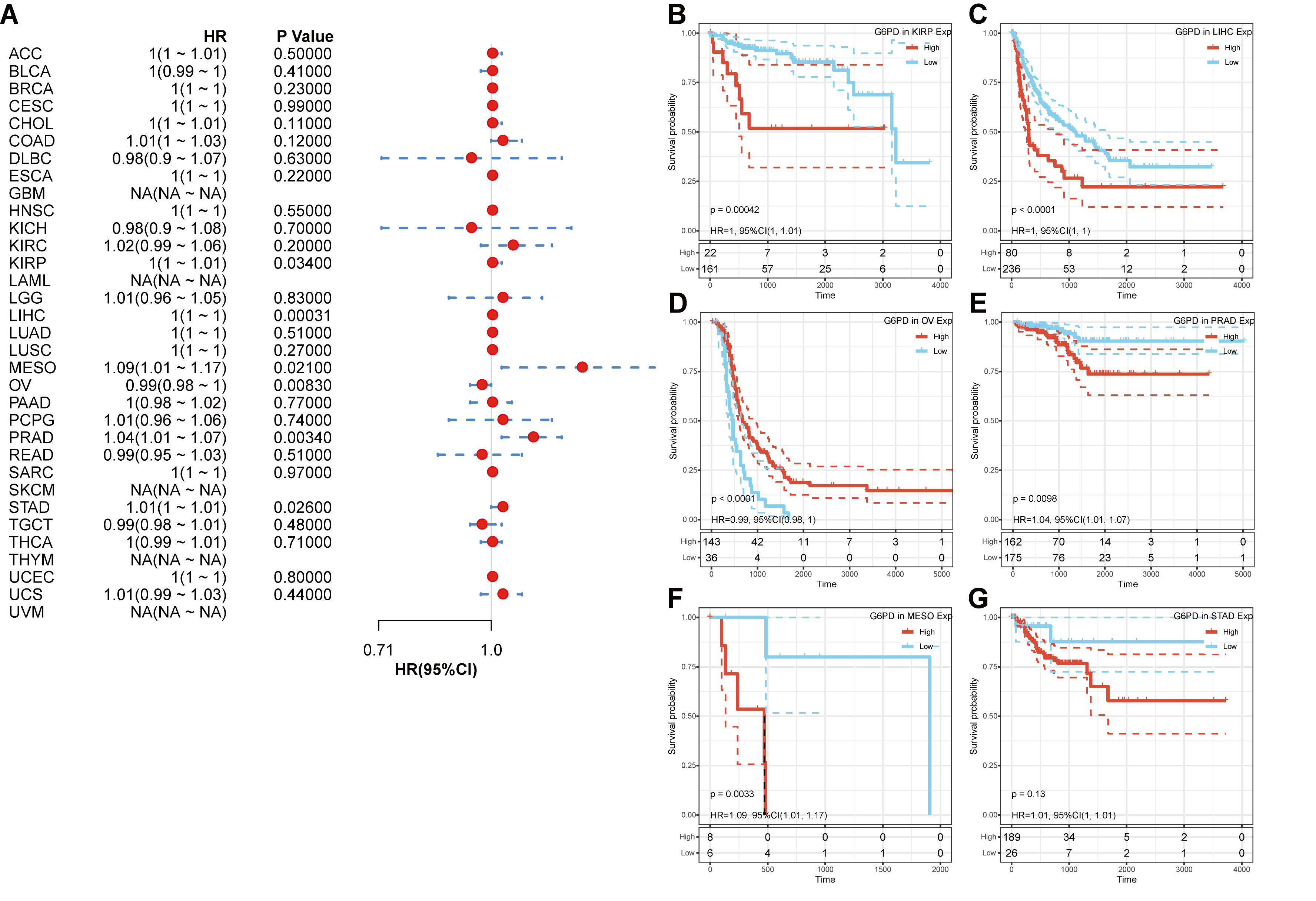

Supplement: Supplementary file 1 [file DataSheet_1.zip › Additional file/Additional file1/Figure S2.tif]

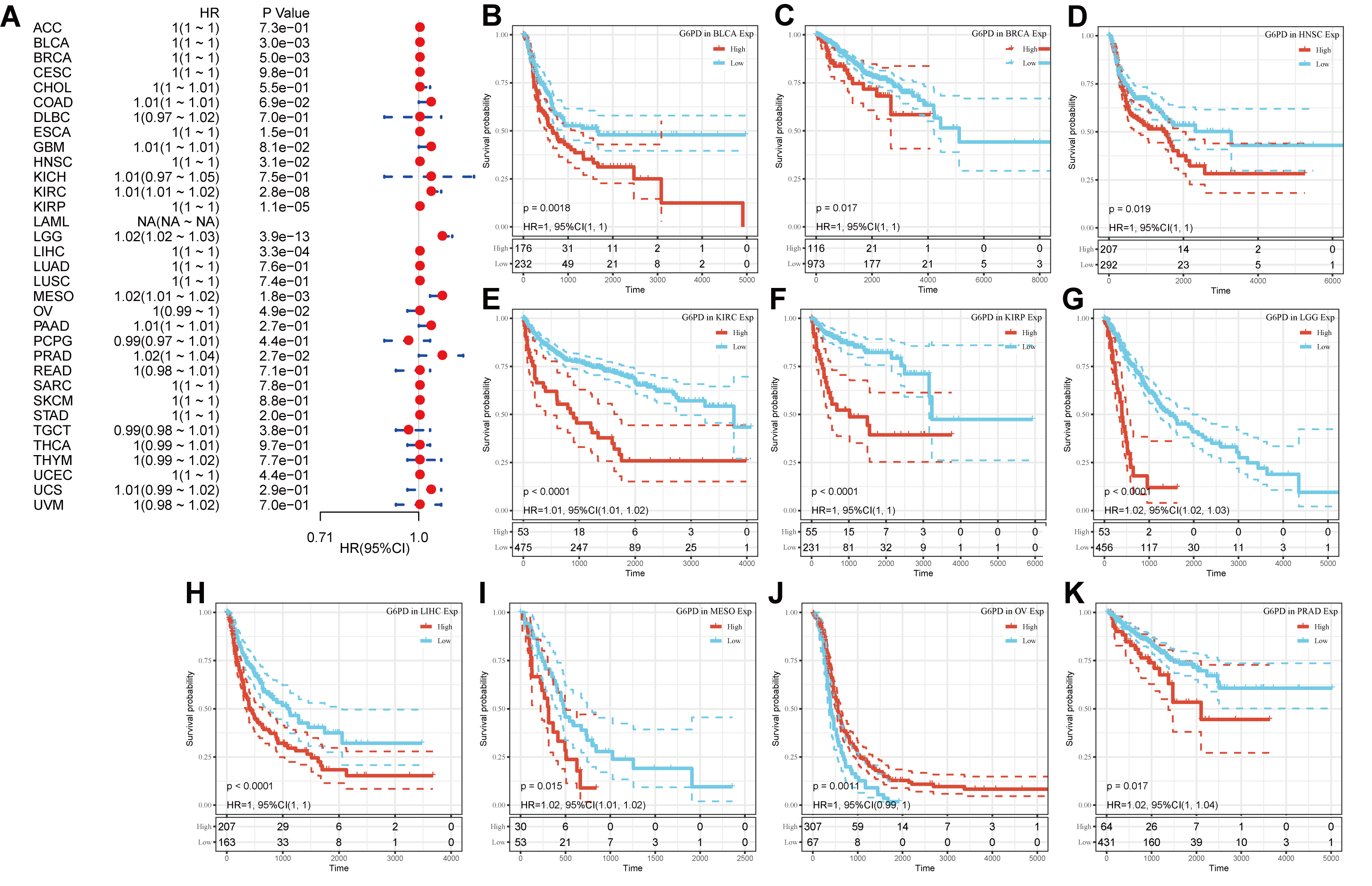

Supplement: Supplementary file 1 [file DataSheet_1.zip › Additional file/Additional file1/Figure S3.tif]
