# Supplementary material for: Pan-cancer analysis reveals that G6PD is a prognostic biomarker and therapeutic target for a variety of cancers
Source: Front Oncol. 2023 Aug 3;13:1183474. doi: 10.3389/fonc.2023.1183474 (PMC10435888; doi:10.3389/fonc.2023.1183474)
Supplement: Supplementary file 1 [file DataSheet_1.zip › Additional file/Description of supplementary material data.doc]

**Figure S1 | Association of G6PD expression with patient disease-specific survival (DSS).**  **(A)** The forest plot shows the relationship of G6PD expression with DSS. **(B-K)** Kaplan-Meier analyses show the association between G6PD expression and DSS.

**Figure S2 | Association of G6PD expression with patient disease-free interval (DFI).** (A) The forest plot shows the relationship of G6PD expression with DFI. (B-G) Kaplan-Meier analyses show the association between G6PD expression and DFI.

**Figure S3 | Association of G6PD expression with patient progression-free interval (PFI). (A)** The forest plot shows the relationship of G6PD expression with PFI. **(B-K)** Kaplan-Meier analyses show the association between G6PD expression and PFI.

**Table S1 | Clinicopathological characteristics of clinical samples and expression of G6PD in LIHC.**

**Table S2 | The complete information of GSEA enrichment analysis**. A total of thirteen gene sets are commonly enriched in the nine prognostic tumors including BLCA, BRCA, HNSC, KIRC, KIRP, LAML, LGG, LIHC, and MESO.

**Table S3 | Detailed drug sensitivity analysis data.** 252 drugs from the GDSC database were significantly associated with G6PD expression in 970 cancer cell lines.
